# The critical role of flow cytometry in diagnosing aberrant T-cell acute lymphoblastic leukemia in a dog: case report

**DOI:** 10.3389/fvets.2026.1793956

**Published:** 2026-07-14

**Authors:** Rafael Costa Bitencourt, Fernanda Regina da Silva, Laura Campos Cassavia Cintra de Oliveira, Gabriel Henrique Crippa, Andrigo Barboza de Nardi

**Affiliations:** 1 Department of Veterinary Clinic and Surgery, Faculty of Agricultural and Veterinary Sciences, São Paulo State University (FCAV/UNESP), Jaboticabal, São Paulo, Brazil; 2 Department of Pathology, Reproduction, and One Health, FCAV/UNESP, Jaboticabal, São Paulo, Brazil

**Keywords:** canine hematology, double-negative phenotype, hematologic malignancy, Imunophenotyping, lymphoblast

## Abstract

Acute lymphoblastic leukemia (ALL) with double-negative T-cell phenotype (CD4-/CD8-) represents a rare presentation in dogs requiring flow cytometry for definitive diagnosis. An eight-year-old German Shepherd presented with progressive lethargy, weight loss, and severe cytopenias. Complete blood count (CBC) revealed severe leukocytosis (90,500/μL) with 99% atypical lymphocytes, non-regenerative anemia, and thrombocytopenia. While blood smear examination demonstrated lymphoblastic morphology, flow cytometry was essential to identify the aberrant immunophenotype CD3+/CD4-/CD8-/CD34+, confirming ALL with arrested differentiation at the earliest T-cell stage. Palliative chemotherapy produced minimal response, and the patient died 11 days post-diagnosis. Flow cytometry results were received 5 days post-mortem, highlighting challenges in turnaround time for specialized diagnostics. This case emphasizes that flow cytometry is essential for accurate diagnosis of acute leukemia with aberrant phenotypes and underscores the need for rapid immunophenotyping services to enable timely therapeutic intervention.

## 1 Introduction

Acute lymphoblastic leukemia (ALL) represents an aggressive hematologic malignancy characterized by uncontrolled proliferation of immature lymphoid precursors in bone marrow with peripheral blood dissemination (1, 2). In dogs, ALL comprises approximately 10–30% of acute leukemias and presents significant diagnostic challenges. Diagnosis requires integration of clinical findings, morphological evaluation, and immunophenotypic characterization to determine lineage and identify prognostically relevant subtypes (3, 4).

Flow cytometry has emerged as the reference standard for immunophenotyping hematologic malignancies, allowing differentiation between T-cell and B-cell origin, identification of aberrant phenotypes, and confirmation of blast cell immaturity through CD34 expression (5, 6). While cytomorphology identifies lymphoblastic features, it cannot reliably distinguish lineage or detect atypical marker expression patterns. Most canine T-cell leukemias express CD3 with either CD4 or CD8 co-receptors (7, 8). However, aberrant immunophenotypes, particularly double-negative (CD4-/CD8-) T-cell ALL, have been increasingly recognized and may represent distinct biological entities (9).

This case demonstrates the essential role of flow cytometry in diagnosing aberrant T-cell ALL. The CD3+/CD4-/CD8-/CD34 + immunophenotype could not have been determined by any other diagnostic modality, illustrating why flow cytometry has become indispensable in veterinary hematopathology. Additionally, this case highlights practical challenges including diagnostic delays when specialized testing is unavailable and consequences of delayed recognition of the underlying hematologic malignancy. The double-negative phenotype suggests arrested differentiation at the earliest committed T-cell precursor stage, contributing valuable documentation to limited veterinary literature.

## 2 Case report

### 2.1 Patient information and clinical findings

An eight-year-old spayed female German Shepherd Dog (40 kg) was presented to a Veterinary Teaching Hospital with a two-week history of progressive lethargy, marked inappetence, and a 5 kg weight loss over 3 weeks. Four weeks earlier, pneumonia had been diagnosed and treated with amoxicillin–clavulanate (20 mg/kg PO q12h) and prednisolone (1 mg/kg PO q24h) for 10 days. Despite initial partial improvement, clinical signs recurred and progressively worsened, leading to referral for further diagnostic evaluation.

Physical examination revealed lethargy, a body condition score of 3/9, mild dehydration (5%), and mild bilateral serous nasal discharge. Peripheral lymph nodes were within normal limits, and no organomegaly was detected on physical examination.

### 2.2 Timeline

Table 1 summarizes the complete clinical timeline with hematological parameters, clinical observations, and therapeutic interventions throughout the disease course.

**Table 1:** The complete clinical timeline with hematological parameters, clinical observations, and therapeutic interventions throughout the disease course.

Parameter	Day 0	Day 3	Day 7	Day 10	Reference range
Hematological data					
WBC (×10^3^/μL)	90.5	88.2	82.1	78.9	8.0–16.0
Lymphocytes (×10^3^/μL)	89.6	87.1	80.8	76.9	0.8–5.1
RBC (×10^6^/μL)	3.4	3.2	3.0	2.8	5.5–7.0
Hemoglobin (g/dL)	9.3	8.7	8.1	7.6	11.0–15.5
Hematocrit (%)	24	21	20	19	34–40
Platelets (×10^3^/μL)	76	68	52	60	175–500
Metarubricytes (%)	3	-	-	-	0
Clinical observations					
Mental status	Lethargic	Alert, improved	Weak, lethargic	Markedly weak	-
Appetite	Poor	Improved, polyphagia	Declining	Anorexic	-
Mucous membranes	Normal	Slightly pale	Pale	Markedly pale	-
Activity Level	Reduced	Transiently increased	Severely reduced	Recumbent	-
Treatment protocol					
Prednisolone	1 mg/kg PO q24h	2 mg/kg PO q24h	2 mg/kg PO q24h	2 mg/kg PO q24h	-
Chlorambucil	-	-	6 mg/m^2^ PO q24h (initiated day 4)	6 mg/m^2^ PO q24h	-
Supportive care	Amoxicillin-clavulanate, Acetylcysteine, Gabapentin	Continued antibiotics and mucolytics	IV fluids, Appetite stimulants added	Intensive supportive care	-

### 2.3 Diagnostic assessment

Complete blood count demonstrated severe leukocytosis (90,500/μL; reference 8,000–16,000/μL) with 89,595/μL atypical lymphocytes (99% of WBCs). Non-regenerative anemia was present with RBC 3.4 × 10^6^/μL, hemoglobin 9.3 g/dL, and hematocrit 24%. Severe thrombocytopenia (76,000/μL; reference 175,000-500,000/μL) was documented. Serum biochemistry showed mild hypoalbuminemia (2.35 g/dL) with normal creatinine and alanine aminotransferase (ALT).

Thoracic radiography (3 incidences) revealed chronic bronchial pattern without active pneumonia or masses. Abdominal ultrasound did not reveal sonographic abnormalities of the liver or spleen, and intra-abdominal lymph nodes showed no enlargement or morphological changes.

**Figure 1:**
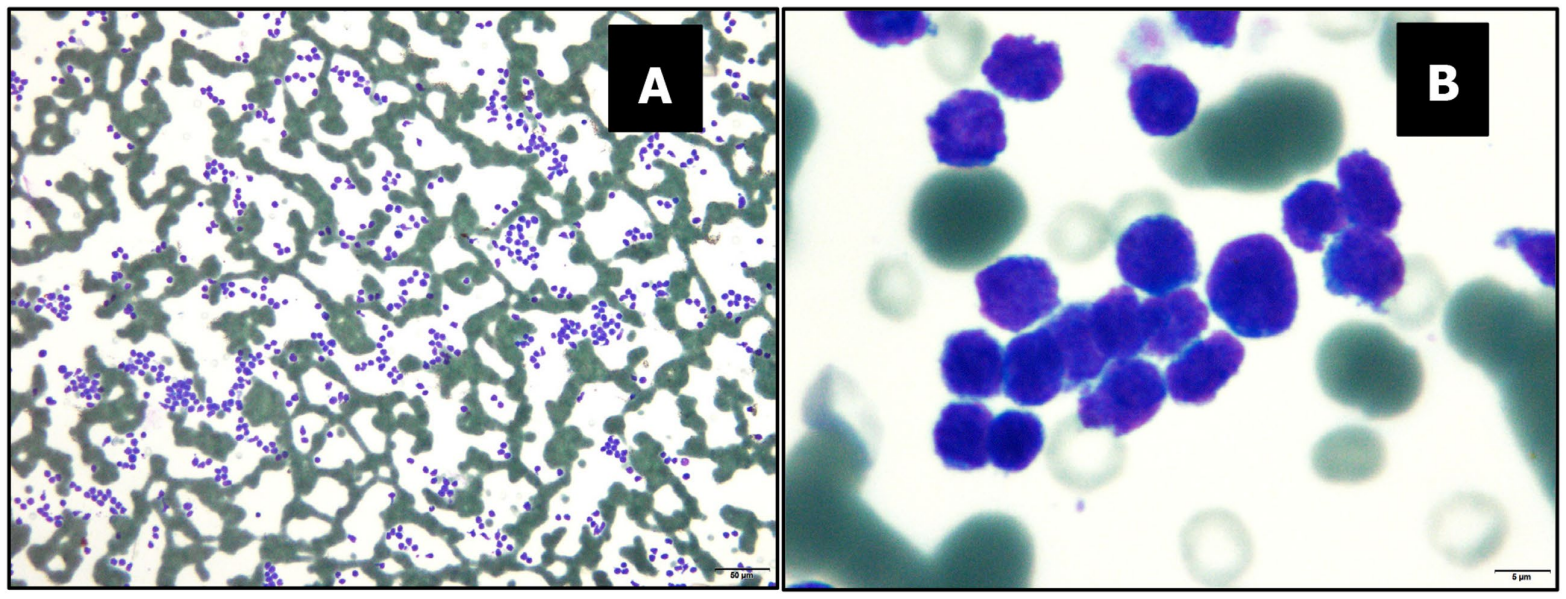
Blood cytology showing marked lymphocytosis with a predominance of intermediate and atypical lymphocytes. **(A)** Low-magnification view (obj. 20×) demonstrating the extensive infiltration of atypical lymphoid cells throughout the smear. **(B)** High-magnification view (obj. 100 × with 2 × optical zoom) revealing cells with lymphoblastic morphology, characterized by marked pleomorphism, loose to coarsely clumped chromatin, prominent nucleoli (1–3 per cell), and azurophilic cytoplasm. The cytological findings were initially suggestive of lymphoblastic lymphoma and were subsequently confirmed as T-cell acute lymphoblastic leukemia by multiparametric flow cytometry. Flow cytometry was performed using peripheral blood collected in EDTA. Cell surface markers were evaluated using conjugated antibodies: anti-canine CD3-APC, CD4-PE, CD8-FITC, CD21-FITC, and CD34-PE. Following erythrocyte lysis, 10,000 events per sample were acquired and analyzed using FlowJo software with automatic compensation. Forward scatter and side scatter analysis identified a distinct population representing 92% of analyzed events with characteristics compatible with immature lymphoid cells (Figure 2).

**Figure 2:**
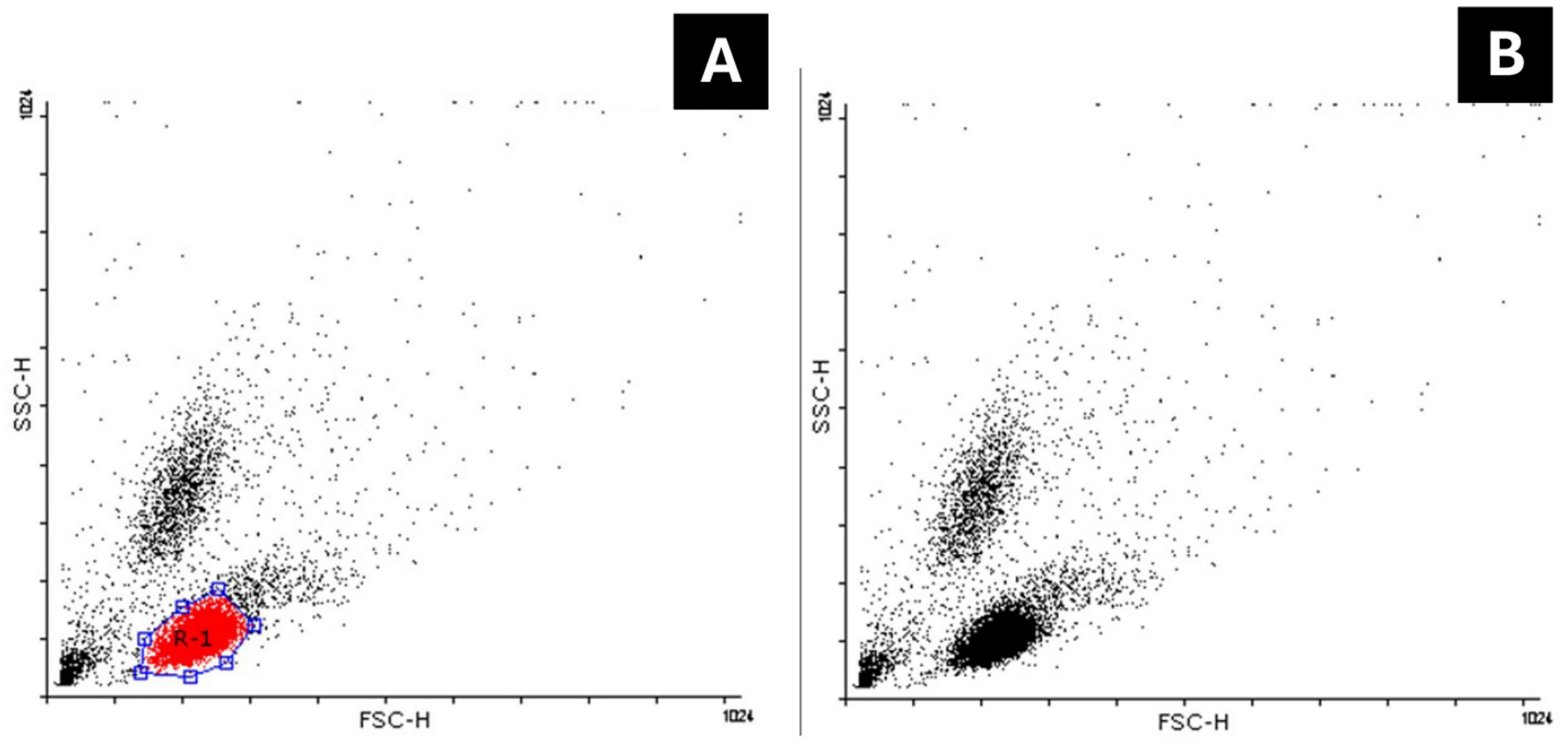
**(A)** FSC-H versus SSC-H scatter plot with gate R1 delineating the population selected for immunophenotyping. **(B)** Ungated cell distribution showing a predominant population with low-to-moderate FSC and low SSC, consistent with immature lymphoid cells.

Blood smear examination revealed monotonous population of intermediate-to-large lymphoid cells with high nucleus-to-cytoplasm ratio, round to indented nuclei, finely stippled to coarsely clumped chromatin, and prominent nucleoli (1–3 per cell). Cytoplasm was basophilic with occasional vacuolation, mitotic figures were occasionally observed, and abundant basket cells were present. Morphology was consistent with lymphoblasts, but lineage determination required flow cytometry (Figures 1, 2).

**Figure 3:**
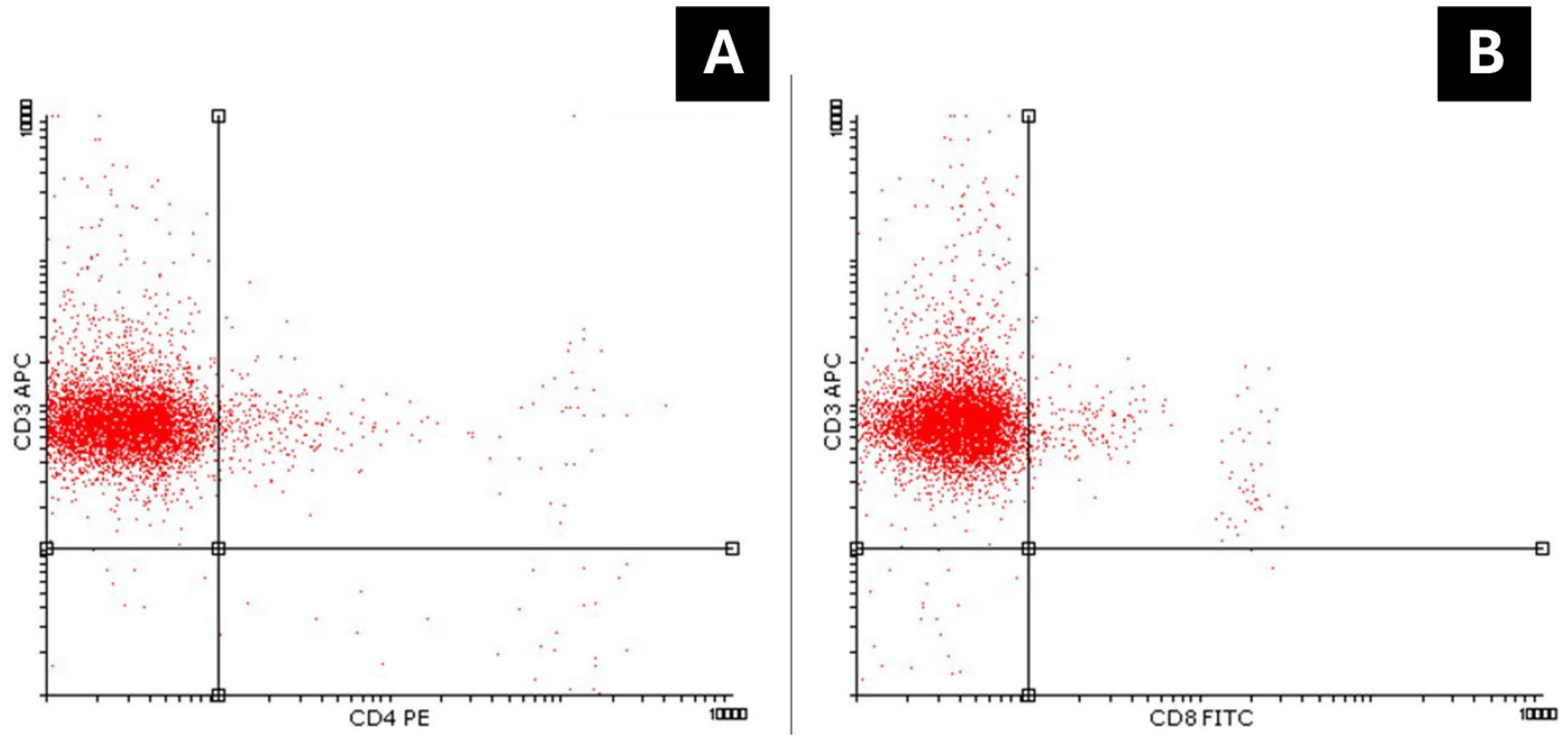
Immunophenotypic analysis by flow cytometry of the gated cell population (R1) from peripheral blood of a dog with acute lymphoblastic leukemia. **(A)** Dot plot of CD3 (APC) versus CD4 (PE) demonstrating a predominant CD3^+^ cell population, consistent with T-cell lineage, with absent or low CD4 expression in most analyzed cells. **(B)** Dot plot of CD3 (APC) versus CD8 (FITC) showing a predominance of CD3^+^/CD8^−^ cells, supporting the diagnosis of T-cell acute lymphoblastic leukemia.

Immunophenotypic analysis demonstrated strong CD3 expression (94.2%), confirming T-cell lineage (Figure 3). Critically, both CD4 (2.1%) and CD8 (1.8%) were negative, defining the aberrant double-negative phenotype (Figure 3). CD34 was expressed in 87.6% of blasts, confirming immature precursor status. CD21 was negative (0.9%), supporting the exclusion of B-cell lineage. The final immunophenotype CD3+/CD4-/CD8-/CD34 + could only be established through flow cytometry.

**Figure 4:**
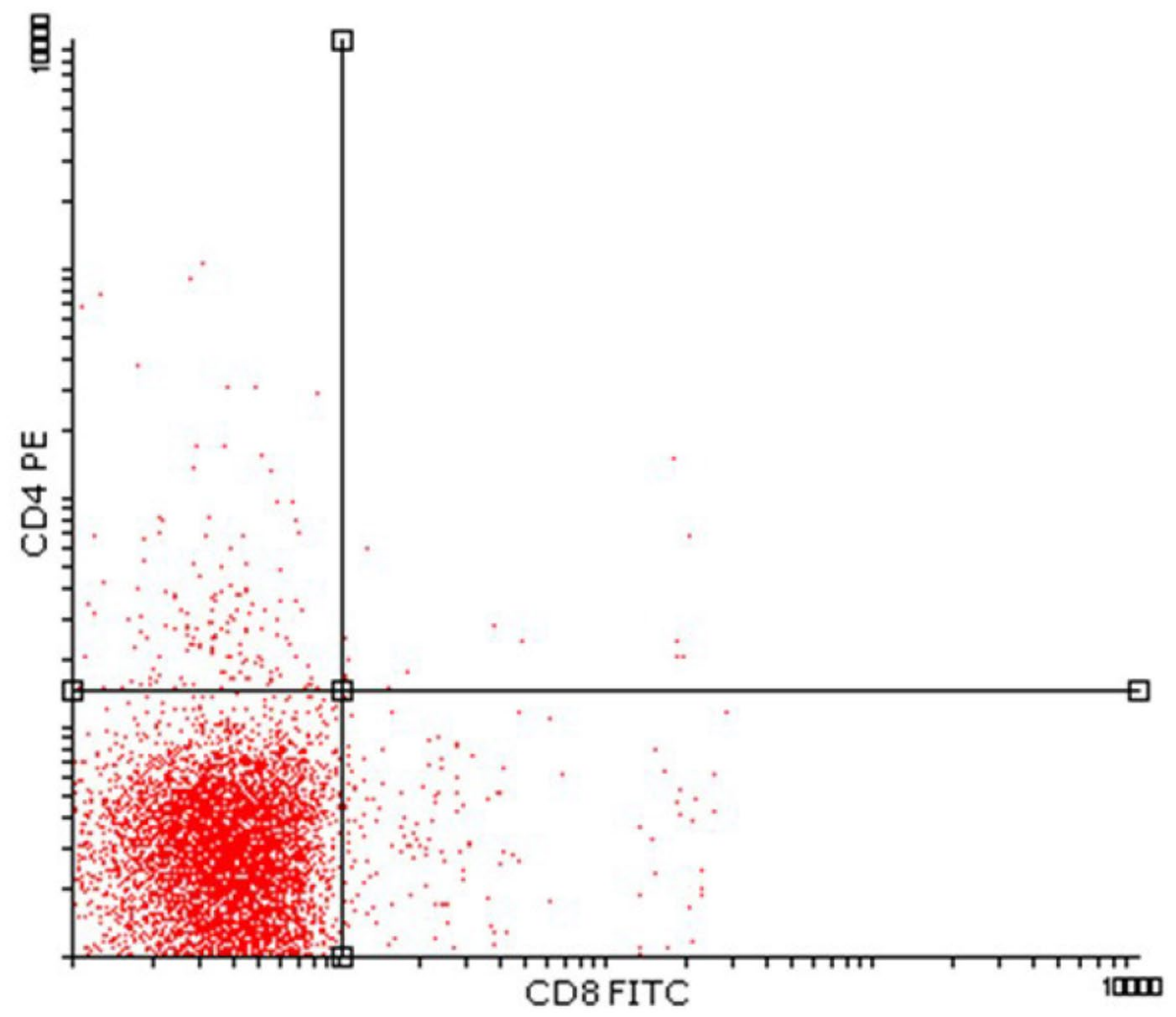
Flow cytometry dot plot of CD4 (PE) versus CD8 (FITC) expression in the gated cell population (R1) from peripheral blood of a dog with acute lymphoblastic leukemia. The majority of analyzed cells lack expression of both CD4 and CD8, resulting in a predominant CD4^−^/CD8^−^ (double-negative) immunophenotype. This finding is consistent with an immature T-cell population and further supports the diagnosis of T-cell acute lymphoblastic leukemia.

Differential diagnoses included chronic lymphocytic leukemia (excluded by CD34-positive phenotype), stage V lymphoma (excluded by absence of organomegaly), B-cell ALL (excluded by CD21-negative, CD3-positive immunophenotype), and acute myeloid leukemia (excluded by CD3-positive expression) (Figure 4).

### 2.4 Therapeutic intervention and outcomes

Initial treatment (days 0–3) targeted presumed chronic bronchitis with amoxicillin-clavulanate, prednisolone (1 mg/kg PO q24h), acetylcysteine, and gabapentin. On day 4, palliative chemotherapy was implemented: prednisolone increased to 2 mg/kg PO q24h and chlorambucil (6 mg/m^2^ PO q24h) initiated. This approach reflected client financial constraints and grave prognosis (median survival 25–50 days even with intensive therapy). Whether the immunophenotypic results had been available at Day 1, the recommended protocol would have been a CHOP-based regimen or L-asparaginase induction; however, the therapeutic decision ultimately reflected shared decision-making between the clinical team and the owner under real-world financial constraints.

As shown in Table 1, treatment response showed minimal lymphocyte reduction (15% over 10 days), progressive anemia (24 to 19% hematocrit), and worsening thrombocytopenia (76,000 to 60,000/μL). Transient improvement occurred days 2–4 following prednisolone escalation, with increased appetite and improved mental status. However, by day 7, progressive weakness and decreased responsiveness developed despite continued chemotherapy. The patient died on day 11 from the date of diagnosis, 7 days after chlorambucil initiation.

The 16-day flow cytometry turnaround time, arriving 5 days post-mortem, prevented phenotype-directed therapeutic adjustments. Survival of 11 days from diagnosis was within the expected range for non-responsive cases, though shorter than reported median estimates, reflecting aggressive disease biology, the inherent limitations of palliative therapy, and the absence of phenotype-directed treatment.

## 3 Discussion

This case suggests that flow cytometry plays a central role in the accurate diagnosis of ALL with aberrant immunophenotypes, particularly when morphological evaluation alone cannot determine lineage. The CD3+/CD4-/CD8-/CD34 + phenotype could not have been determined by cytomorphology, clinical presentation, or any other modality. While blood smear examination revealed lymphoblastic morphology, only flow cytometry provided definitive T-cell lineage determination, identified absent CD4/CD8 co-receptors, confirmed CD34 expression strongly associated with acute leukemia and helping distinguish it from chronic leukemia, and excluded B-cell lineage through absent CD21 expression (5).

The double-negative phenotype has important biological significance. In normal thymic development, lymphoid progenitors progress through double-negative (CD4-/CD8-), double-positive (CD4+/CD8+), and finally single-positive mature stages (8). While CD4-/CD8- negativity is a recognized step in normal thymic ontogeny, the concurrent expression of surface CD3 (a marker of T-cell commitment) alongside CD34 (a primitive hematopoietic precursor marker) in circulating leukemic blasts is immunophenotypically atypical. In normal thymic development, early DN (double-negative) thymocytes do not yet express surface CD3, and CD34 + precursors are not found circulating in peripheral blood at high numbers. The co-expression of CD3 and CD34 in peripheral leukemic blasts therefore represents arrested differentiation at an early but partially committed T-cell precursor stage, rather than a simple DN phenotype. This phenotype is relatively uncommon in canine T-cell malignancies; the CD8 + T-cell phenotype predominates in canine chronic lymphocytic leukemia (CLL), while T-cell ALL more commonly expresses CD4 or CD8 co-receptors (7, 9).

In humans, double-negative T-ALL represents approximately 5–10% of cases and associates with specific genetic aberrations and variable prognosis (10, 11). Within human immunophenotypic classification systems, the CD3+/CD4-/CD8-/CD34 + phenotype is most analogous to early T-precursor ALL (ETP-ALL), characterized by incomplete T-lineage commitment and generally aggressive biological behavior. Whether similar implications exist in canine double-negative T-ALL remains unknown due to limited studies correlating immunophenotype with clinical outcome (11, 12).

CD34 expression (87.6%) was strongly associated with acute leukemia and helped distinguish it from chronic lymphoproliferative disorders. CD34, a hematopoietic stem and progenitor cell marker, is common in ALL but rare in mature lymphoid neoplasms (13, 14). Without flow cytometric CD34 assessment, definitive classification would have been uncertain. Similarly, absent CD21 expression supported the exclusion of B-cell lineage, which cannot be reliably determined by morphology alone.

A critical limitation was the 16-day turnaround time for flow cytometry results. The blood sample was collected in EDTA on Day 1 and shipped via courier to an external reference laboratory in Brazil on the same day. The delay reflected a combination of shipping logistics, laboratory processing queue, and reporting time, a constraint specific to the regional veterinary diagnostic infrastructure rather than a universal benchmark. To our knowledge, sample quality was deemed adequate by the laboratory at the time of analysis.

This delay prevented phenotype-directed therapeutic adjustments and highlights an important practical challenge: while flow cytometry is essential for diagnosis, its clinical utility diminishes when results are unavailable to influence treatment decisions in rapidly progressive diseases. In this specific patient, the flow cytometry result had no impact on clinical management, as it was received 5 days after death. Its value lies exclusively in the documentation of an immunophenotypically rare case. This illustrates urgent need for rapid turnaround immunophenotyping services. In human medicine, flow cytometry results are typically available within 24–48 h, enabling prompt risk-stratified therapy initiation. The veterinary community should strive to achieve similar turnaround times. In Brazil, veterinary flow cytometry remains largely centralized in a small number of reference laboratories, with no established satellite network for rapid immunophenotyping. Advocacy for expanded access to decentralized services is warranted.

Despite delayed confirmation, empirical treatment was appropriately initiated on day 4 based on morphologic suspicion. However, the palliative protocol with prednisolone and chlorambucil produced minimal response. The poor outcome likely reflects a combination of factors: the palliative nature of the chosen protocol (constrained by financial limitations), the patient’s advanced age and severely compromised clinical status at presentation, the prior corticosteroid exposure potentially inducing glucocorticoid resistance, and the possibility that the aberrant double-negative T-cell phenotype itself confers inherent biological aggressiveness. The disease may have followed a refractory course regardless of treatment intensity. Standard intensive protocols like CHOP offer superior remission rates, but even with aggressive chemotherapy, median survival for canine ALL remains dismal at 25–50 days (4, 15, 16).

The initial clinical presentation mimicked lower respiratory tract disease, leading to symptomatic management prior to referral. This represents an important teaching point, as non-specific systemic signs may delay recognition of underlying hematologic malignancy. Furthermore, prior corticosteroid administration may have partially masked the leukemic burden by inducing transient lymphocytolysis and potentially altering the morphological appearance of the circulating neoplastic cells. This underscores the critical importance of performing CBC in dogs with systemic illness, particularly with weight loss or poor body condition, as part of a comprehensive diagnostic workup integrating clinical history, physical examination, and advanced diagnostics as indicated. The severe pancytopenia pattern reflects bone marrow replacement by neoplastic cells, a hallmark prompting immediate hematologic investigation (1, 2).

The early detection of marked leukocytosis with atypical lymphocytosis on CBC, integrated with clinical history and physical examination, could have represented an opportunity for earlier hematologic screening and referral. However, the complexity of aberrant immunophenotypes in ALL underscores that early recognition alone does not guarantee a straightforward diagnostic or therapeutic course.

Prior corticosteroid administration represents a confounding factor, as prolonged exposure can alter disease presentation while inducing glucocorticoid resistance that negatively impacts chemotherapy response (4). Whether earlier recognition might have improved outcome remains speculative given the inherently grave prognosis, but this reinforces the importance of thorough diagnostic investigation before initiating empirical corticosteroid therapy.

Bone marrow aspiration was not performed since peripheral blast count of 99% met diagnostic criteria without requiring marrow evaluation (6, 17). However, a marrow biopsy could have provided additional information regarding the degree of myelophthisis, residual hematopoietic reserve, and the microenvironmental context of the disease. Additionally, abdominal organs infiltration was assessed solely by ultrasound, without cytological or histological confirmation through fine needle aspiration (FNA) or biopsy, which represents a diagnostic limitation, as imaging alone cannot definitively exclude neoplastic involvement.

This case also lacked genetic characterization, cytogenetic analysis, and clonality confirmation via PARR (PCR for Antigen Receptor Rearrangements), and minimal residual disease assessment, all of which could have provided additional insights. Future studies incorporating comprehensive immunophenotyping combined with cytogenetic analysis and standardized treatment protocols are needed to better understand biological heterogeneity of canine ALL and identify potential prognostic subgroups.

## 4 Conclusion

This case demonstrates that flow cytometry plays an irreplaceable role in diagnosing acute lymphoblastic leukemia with aberrant immunophenotypes. The CD3+/CD4-/CD8-/CD34 + double-negative phenotype could not have been identified by cytomorphology, clinical findings, or any other modality. Only flow cytometry provided definitive T-cell lineage determination, identified absent CD4/CD8 co-receptors indicating arrested early-stage differentiation, confirmed CD34 expression strongly associated with acute from chronic leukemia, and supported the exclusion of B-cell lineage.

The delayed turnaround time, arriving 5 days post-mortem, rendered the result clinically inutile for this individual patient, and prevented phenotype-directed therapeutic adjustments. This highlights the urgent need for rapid turnaround immunophenotyping services in veterinary medicine. Despite palliative chemotherapy, the patient survived only 11 days from diagnosis (approximately 7 weeks from first clinical signs), consistent with grave prognosis associated with canine ALL.

Veterinary clinicians must maintain high suspicion for hematologic malignancies in dogs presenting with unexplained cytopenias and should prioritize early CBC evaluation, integrated with clinical history and physical examination, as part of a comprehensive diagnostic workup. When acute leukemia is suspected based on blood smear examination, flow cytometric immunophenotyping should be pursued urgently to enable accurate diagnosis and timely therapeutic intervention. This case underscores that modern veterinary hematopathology requires access to flow cytometry for definitive diagnosis of complex hematologic malignancies, and the veterinary community should advocate for expanded availability of rapid turnaround immunophenotyping services.

## Data Availability

The raw data supporting the conclusions of this article will be made available by the authors, without undue reservation.
